# ETosis: A Microbicidal Mechanism beyond Cell Death

**DOI:** 10.1155/2012/929743

**Published:** 2012-02-26

**Authors:** Anderson B. Guimarães-Costa, Michelle T. C. Nascimento, Amanda B. Wardini, Lucia H. Pinto-da-Silva, Elvira M. Saraiva

**Affiliations:** ^1^Instituto de Microbiologia Paulo de Góes, Universidade Federal do Rio de Janeiro (UFRJ), 21941-901 Rio de Janeiro, RJ, Brazil; ^2^Instituto de Biofísica Carlos Chagas Filho, Universidade Federal do Rio de Janeiro (UFRJ), 21941-901 Rio de Janeiro, RJ, Brazil; ^3^Instituto de Veterinária, Universidade Federal Rural do Rio de Janeiro (UFRRJ), 23890-000 Seropedica, RJ, Brazil

## Abstract

Netosis is a recently described type of neutrophil death occurring with the release to the extracellular milieu of a lattice composed of DNA associated with histones and granular and cytoplasmic proteins. These webs, initially named neutrophil extracellular traps (NETs), ensnare and kill microorganisms. Similarly, other cell types, such as eosinophils, mast cells, and macrophages, can also dye by this mechanism; thus, it was renamed as ETosis, meaning death with release of extracellular traps (ETs). Here, we review the mechanism of NETosis/etosis, emphasizing its role in diseases caused by protozoan parasites, fungi, and viruses.

## 1. Introduction

Upon inflammation, neutrophils are the first cells to be recruited to the inflammatory site, in a process orchestrated by chemokines, a series of attractive molecules produced locally. Neutrophils then migrate to the inflamed site where they contain and eliminate microorganisms using three basic strategies: phagocytosis, with ingestion and killing of microorganisms inside special compartments of the cell, degranulation, which consists in the extravasation of the granules content to the extracellular milieu, and by a new antimicrobial mechanism named netosis that also occurs in the extracellular milieu, when DNA associated to proteins is expelled from the cell [1–3]. 

Neutrophils are the most abundant leukocytes in the blood and constitute the first line of host defense against invading pathogens. These cells are also known as polymorphonuclears (PMNs) or granulocytes, since they have a segmented nucleus with lobules linked by nuclear filaments and hold large numbers of three different types of granules. These granules were classified according to their protein content as primary, azurophil, or peroxidase-positive granules because of their high myeloperoxidase (MPO) loading. Besides MPO, these granules possess cathepsin G, defensins, elastase, and proteinase 3, among many others proteins. MPO production stops between promyelocyte and myelocyte transition stages during maturation at the bone marrow, and then, the next granules formed are all peroxidase negative. The secondary granules contain collagenase, gelatinase, lactoferrin, and sialidase, and the tertiary granules enclose gelatinase, *β*2-microglobulin, and others. In addition, secretory vesicles are also present in the neutrophil cytoplasm; however, the protein content of these vesicles has not been completely elucidated [4, 5].

## 2. NETosis Occurs with Extrusion of Neutrophil Extracellular Traps (NETs)

In a seminal work, Brinkmann and colleagues in 2004 described that upon activation by phorbol myristate acetate (PMA), lipopolysaccharide (LPS), interleukin 8 (IL-8), or by Gram-positive and -negative bacteria, neutrophils release their chromatin to the extracellular medium, associated with different proteins, forming the so-called NETs. Currently, there is an increasing list of synthetic and physiological molecules, as well as microorganisms and their products, which can activate neutrophils to release NETs (Table 1).

Initially, NET composition was described as being formed by decondensed chromatin scaffolds associated to proteins of the different types of neutrophil granules [1]. Nicotinamide adenine dinucleotide phosphate (NADPH) oxidase subunits, human peptidoglycan protein short-S, and pentraxin (PTX)-3 were also detected in association with NETs [20, 54, 55]. Later, using proteomic analysis, besides histones and granule proteins, cytosolic and cytoskeleton proteins, catalase and glycolytic enzymes were found linked to the NETs [15]. This proteomic study evidenced that among the NET-associated proteins, histones were the most abundant, followed by the neutrophil elastase. Intriguingly, NADPH oxidase, PTX3, and cathelicidin LL-37 previously detected in NETs by immunofluorescence staining were not found associated to NETs by proteomic analysis [15]. These discrepancies could be due to a loose association of these missing proteins to the NETs, which could have been lost by the NET processing for the proteomic analysis. Recently, IL-17 was found associated to NETs in psoriasis skin biopsies evidenced with specific antibodies by immunofluorescence staining [32].

## 3. NETosis Mechanism

**Figure 1:**
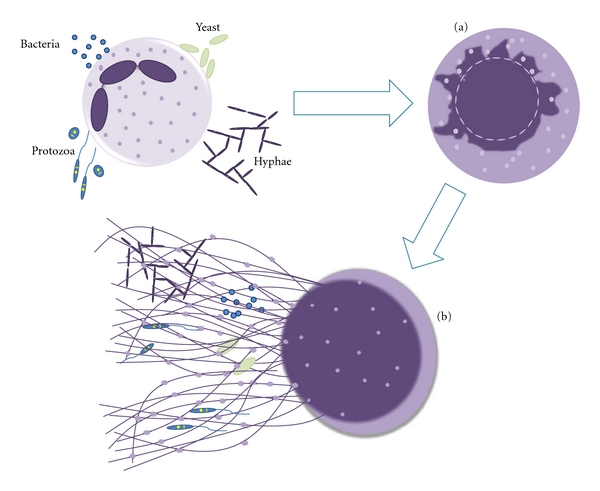
Mechanism of Neutrophil Extracellular Traps release. Neutrophils are stimulated by contact with bacteria, protozoan, fungi (yeast and hyphae forms) or their products (not shown), leading to: (a) ultrastructural alterations of nuclear shape with chromatin decondensation, swollen and fragmentation of the nuclear membrane, which allow the association of granules and cytoplasmic proteins with the chromatin, and (b) release of extracellular structures consisting of a DNA-backbone, decorated with histones, neutrophil granular and cytoplasmatic proteins (NETs), which ensnare and kill microorganisms.

Although very little is known about the mechanism of NET release, some morphological features are easily observed during netosis (Figure 1). Thus, after stimulation, neutrophil nuclei lose segregation of eu- and heterochromatin, its characteristic lobular form vanishes, and the nuclear membrane swells up, fragmenting into vesicles. This is followed by the granules' membranes disruption, which allows the mixing of nuclear, cytoplasmic, and granular contents. Subsequently, the plasma membrane is permeabilized, allowing NET release to the extracellular milieu [22]. NET structure is composed of smooth strands of 15–17 nm diameter decorated with globular domains ranging from 25 to 50 nm [1, 15].

 Netosis is a new type of cell death, different from necrosis and apoptosis [22]. Under netosis, there is neither DNA fragmentation nor phosphatidylserine exposure in the outer membrane leaflet, hallmarks of these latter forms of cell death, respectively. The intact nuclear envelope differentiates necrosis from netosis, and NET-DNA was not detected in the culture supernatants of apoptotic or necrotic neutrophils [22]. Moreover, netosis seems to be independent of caspases and RIP-1 kinases, since pretreatment of neutrophils with zVAD-fmk or necrostatin-1 did not affect netosis completion [15, 56]. All these features differentiate netosis from apoptosis and necrosis. However, it was recently shown that netosis induced by PMA was dependent on a simultaneous activation of both autophagy and superoxide production, and neither mechanism isolated was capable to induce netosis [56]. Thus, inhibition of autophagy prevents chromatin decondensation and netosis, without affecting superoxide production, demonstrating that NADPH oxidase activity is necessary, but insufficient to, alone, trigger netosis. Furthermore, induction of autophagy in neutrophils from chronic granulomatous disease patients, which possess a defective NADPH oxidase, thus, unable to generate reactive oxygen species (ROS), was not sufficient to promote chromatin decondensation [56].

In addition, netosis seems to occur by a mechanism independent of neutrophil granule exocytosis, since Rab27a-deficient neutrophils, which are deficient in exocytosis, are able to release NETs [20].

Presently, the great majority of stimuli described to induce netosis are dependent on ROS production by the multienzyme complex NADPH oxidase. Drugs that inhibit NADPH enzyme, hence, inhibit NET release [22, 44, 57]. Moreover, neutrophils from patients with chronic granulomatous disease are incapable of forming NETs [22, 58, 59].

However, a novel netosis process was recently described for the bacterium *Staphylococcus aureus*, occurring independently of ROS and neutrophil lysis [45]. In this new mechanism of netosis, that occurs very rapidly, vesicles containing DNA sprout from the nuclear membrane are extruded intact to the extracellular environment, where they break and release their chromatin content that traps and kills bacteria. This more rapid mechanism would precede the ROS-dependent NET release [45]. Similarly, ROS generation is not sufficient to rescue netosis from patients suffering from syndrome of neonatal neutrophil dysfunction, a disease associated with sepsis and other severe infections [57]. Another ROS-independent NET release was reported for *Leishmania donovani *[37].

It has been demonstrated at the molecular level that chromatin decondensation, a pivotal event during netosis, occurs with elastase migration from the primary granules to the neutrophil nuclei, where this enzyme partially degrades histones [33]. As follow, myeloperoxidase also migrate to the nucleus, synergizing with elastase for the chromatin decondensation by a still unknown mechanism, which is independent of its enzymatic activity. Importantly, neutrophils from myeloperoxidase-deficient patients are unable to release NET [60]. Treatment of neutrophils with specific neutrophil-elastase inhibitors abrogates NET formation, and purified neutrophil elastase was able to digest histones and promote chromatin decondensation in isolated nuclei. Moreover, mice knockout for the elastase-encoding gene did not produce NET in a model of pulmonary infection by *Klebsiella pneumoniae* [33].

Another important event involved in chromatin decondensation is histone hypercitrullination, a reaction catalyzed by peptidyl arginine deiminase 4 (PAD4), in which histones' arginines are converted to citrullines by deimination. Hypercitrullinated histones were detected in NETs, but not in apoptotic neutrophils [11, 61]. It has also been demonstrated that mice knockout for PAD4 enzyme are unable to form NETs upon activation by different stimuli, being deficient in bacterial killing by these traps [11, 62, 63]. Infection of PAD^+/+^ and PAD^−/−^ mice with group A *Streptococcus* confirmed that PAD^−/−^ animals were more susceptible to infection, presenting more lesions than PAD^+/+^. Moreover, neutrophils purified from PAD^−/−^ animals did not release NETs when stimulated by lipopolysaccharide or oxygen peroxide as their wild-type counterparts [62].

Hitherto, we know that upon protein kinase C activation by PMA or by diacylglycerol (DAG) analogs, Raf-MEK-ERK pathway is required for NET formation and also that this signaling pathway is upstream of NADPH oxidase activation, since diphenylene iodonium (DPI) did not abolish phosphorilation of ERK [27]. Moreover, the antiapoptotic protein myeloid cell leukemia (Mcl)-1 is overexpressed in PMA-activated neutrophils, a pathway required for PMA/DAG/*Helicobacter pylori* NET induction [27].

The participation of Rac2, an isoform of Rac small GTPases, in NET induction by PMA or LPS-stimulated mice neutrophils has also been evidenced [44]. Rac2 null mice have negligible NET production in comparison to Rac1 null and wild-type counterparts, showing that Rac2 isoform is required for NET release. Rac2 mutants are unable of producing NETs due to a lack of ROS production, which is rescued by the addition of hydrogen peroxide to neutrophils. Since Rac has been shown to regulate nitric oxidase synthase (NOS), the role of nitric oxide (NO) on NET production by wild-type and Rac2 mutant mice was investigated. Indeed, L-NAME, an NOS inhibitor, reduced NET release induced by PMA in mice neutrophils [44]. Confirming the role of nitric oxide on NET production, 7-NI, another NOS inhibitor, also decreased NET release induced by PMA in human macrophages [43]. Intriguingly, treatment with SNAP, an NO donor, did not induce NET release either in wild-type or Rac2 null neutrophils. However, increased NET formation was observed by SNAP treatment of PMA-activated wild-type neutrophils, suggesting that ROS production by NADPH oxidase may be required for NET induction by NO [44]. These discrepancies could be due to the source of the neutrophils used in each study: SNAP induced netosis in human blood neutrophils [43] but not in mice bone marrow neutrophils [44]. 

The participation of NO in NET induction was also demonstrated using SNAP and SNP, two NO donors [43]. Human neutrophils treated with these compounds release NET in a dose- and time-dependent manner, which was inhibited by N-acetyl cysteine, an ROS scavenger. SNAP-induced NET was abolished by DPI, an NADPH oxidase inhibitor, as well as by 4-aminobenzoic acid hydrazide (ABAH), a myeloperoxidase inhibitor, suggesting that NET induction by NO occurs with increasing free radicals generation [43].

## 4. Functions of Neutrophil Extracellular Traps

 NETs grab microbes avoiding their dissemination through the organism, and also offering a high local concentration of antimicrobial proteins. Bactericidal activity of NET-associated histones has been proved against *Shigella flexneri*, *Salmonella typhimurium, Salmonella enterica, Staphylococcus aureus, Streptococcus pyogenes, *and *Bacillus anthracis *[1, 64]; however, it remains to be determined whether the bactericidal activity of histones is modulated by its hypercitrullination. In addition, the cytosolic calprotectin protein complex (S100A8/A9) associated to NETs kills *Candida albicans *and *Cryptococcus neoformans* [15].

Net-associated histones and calprotectin have been considered as bactericidal and fungicide, respectively, but many other NET-linked components are also endowed with microbicidal properties, at least in their free forms [1, 65, 66]. These molecules include cathelicidin LL-37, defensins, bacterial permeability-increasing protein (BPI), lactoferrin, myeloperoxidase, proteinase 3, and elastase [3, 4, 67]. Furthermore, these components could act synergistically as shown for two of them, the peptidoglycan recognition protein-S and lysozyme, which colocalize in NETs [67].

Even though resistant to NET-mediated killing, group A *Streptococcus, S. pneumoniae*, *Mycobacterium tuberculosis, *and *Haemophilus influenza *are caught by NETs, suggesting that this property could also have an important role for the host immune response [25, 26, 42].

Presently, it is unknown how so diverse and different microorganisms are ensnared by NETs. Many of the NETs constituents are highly cationic, and it is likely that NETs can bind negatively charged surfaces, while NET-specific recognition sites for microorganisms could not be excluded [68].

Albeit NETs are toxic for microorganisms, some microbes are able to escape the NET-mediated killing. To name a few of these strategies, the capsule expression and M1 protein of group A *Streptococcus* are important to NET resistance [69]. In addition, endonucleases expressed by *Staphylococcus*, *Streptococcus pneumonia,* and group A *Streptococcus* enhance bacterium survival by digesting NET-DNA scaffold [34, 49, 70–72]. Importantly, since DNA constitutes the NET backbone, its digestion with DNase rescues microorganisms from NET-mediated killing [1, 13, 35]. NET-escape mechanisms as well as NET role on autoimmune diseases are reviewed elsewhere [13, 73–77].

## 5. Cells Able to Release Extracellular Traps (ETosis)

Netosis was first described in neutrophils, thus the origin of its name. However, other cells such as eosinophils and mast cells also release extracellular traps (ETs) composed by DNA and antimicrobial proteins [28, 30]. Monocytes and macrophages were also shown to release extracellular traps, but to a lesser extent when compared to neutrophils, and the microbicidal activity of these ETs has not yet been determined [21, 78]. Since it seems to be a more general mechanism, shared by different cell types, the release of intracellular DNA to the extracellular milieu was renamed as ETosis, meaning death with release of DNA extracellular traps. Interestingly, eosinophils release their mitochondrial DNA, in a death-independent way [30]. These same authors reported that GM-CSF-primed neutrophils released mitochondrial DNA associated with granule proteins, forming neutrophil extracellular traps in response to LPS or complement C5a fragment [24].

Besides occurring in different cell types, ETosis seems to be a well-conserved mechanism, since release of ETs was reported for neutrophils or related cells in many different organisms, such as ox, horse, fish, mouse, and cat neutrophils, as well as chicken heterophils [12, 17–19, 29, 36, 38, 39, 44]. Although classical ETs were not observed in *Galleria mellonella*, DNA derived from oenocytoid cells participates in the haemolymph coagulation, an important mechanism for microbes killing in insects [79].

Interestingly, ETs were also evidenced in plants and seem to play an important role on root tip defense against fungal infections, implying a similar behavior between plant root and animal cells, which extrude ETs in a defense mechanism important for plant growth and survival [80, 81].

Because it was first described in neutrophils, which are easy cells to work with, the majority of the data available analyze netosis aspects, mainly when the studies were related to infectious diseases, although it has an important role in autoimmune diseases such as systemic lupus erythematosus and vasculitis [75–77].

## 6. NETs and Protozoa

**Figure 2:**
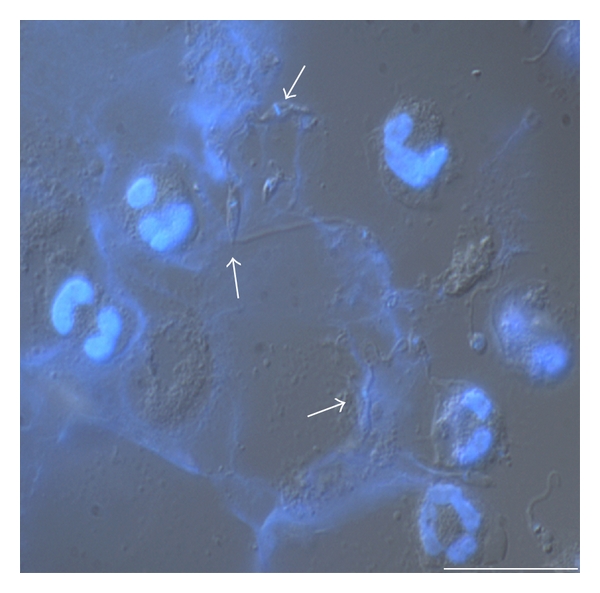
Immunostaining of NETs induced by *Leishmania*. Naïve neutrophils were incubated with promastigotes (1 : 5 ratio) for 1 h at 35°C. Cells were fixed and stained with DAPI and shown merged with differential interference contrast image. Arrows point to NET-ensnared promastigotes (Bars: 20 *μ*m).

**Figure 3:**
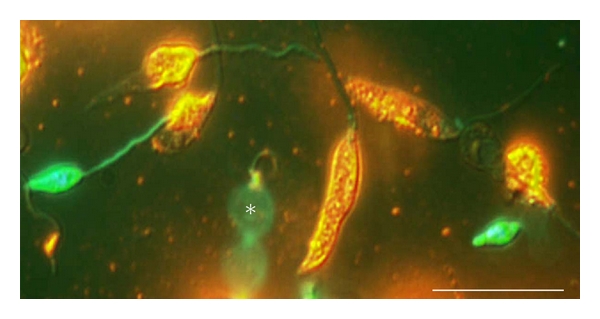
Histone toxicity to promastigotes. Promastigotes were incubated with purified histone for 30 min and stained by the live/dead method. Dead promastigotes stained in yellow/orange and live promastigotes in green. Differential interference contrast image merged with the fluorescence staining of the same cells. (*) The beating of a live promastigote flagellum. Bars, 20 *μ*m.

Neutrophils are rapidly recruited to infection sites during infections with many protozoa and microbial pathogens, pointing out the importance of these phagocytes in the immune response to these infectious agents. *Leishmania* particularly meets neutrophils in the very beginning of the infection process, since these protozoan parasites are inoculated by the sand fly vector in a pool of blood. Thus, based on the interaction between *Leishmania* and neutrophils, we investigate the capacity of this trypanosomatid to induce netosis, to better understand the first steps of the *Leishmania *infection [35]. Initially, we detected that both forms of the parasite, promastigotes and amastigotes, were able to induce NET extrusion in human neutrophils and were caught by the resulting meshes (Figure 2; see Supplementary video in supplementary material available online at doi:10.1155/2012/929743). In addition, we demonstrated that the glycoconjugate lipophosphoglycan, which is expressed on the promastigote cell surface, induced neutrophils to release NET in a dose-dependent manner. NETs were toxic for the promastigotes, an effect mediated by the histones present in the lattices, whose toxicity to the parasite was neutralized by antihistone antibodies [35]. The ability of histones to kill *Leishmania *was further confirmed by parasite death upon its exposure to histones purified from calf thymus (Figure 3). NETs occurrence goes further beyond experimental findings since the presence of NETs in lesion biopsies of patients with active cutaneous leishmaniasis was also shown [35]. 

*Leishmania *sensitivity to histone-mediated toxicity also includes promastigotes of *L. mexicana, L. braziliensis, L. major, and L. amazonensis*, although the death mechanism mediated by these proteins was still unknown [82]. Interestingly, amastigotes from *L. mexicana *and *L. amazonensis* were resistant to the histone-mediated toxicity [82]. NET induction was also shown for *L. donovani *promastigotes, but, contrary to the former findings, the authors reported that both *L. donovani *and *L. major* promastigotes were resistant to the NET-mediated killing [37].

Apicomplexans such as *Plasmodium*, the causative agent of malaria, and *Eimeria,* the causative agent of eimeriosis in cattle, comprise some of the most life-threatening protozoan parasites. Although a direct NET induction by *Plasmodium* has not been described yet, there is one report showing NETs in the blood of *Plasmodium falciparum*-infected children, showing infected erythrocytes and trophozoites attached to structures identified as NET by DNA staining [83].

Behrendt and coworkers [16] reported that sporozoites of *Eimeria bovis* induce NET extrusion by calf neutrophils, and this traps snare sporozoites. Although a NET-lethal effect on *Eimeria* sporozoites was not demonstrated, the parasite infectivity to bovine primary endothelial cells was decreased in comparison to untreated parasites. Thus, arrest of *Eimeria* sporozoites by NETs may prevent host cell invasion required for this parasite replication.

## 7. NETs and Fungi

Fungal pathogens cause a wide and increasing number of severe infections with high mortality rates, mainly in immunocompromised individuals. The phenomenon of NET release was observed in experimental models of fungal infections using *Candida albicans, Cryptococcus neoformans, Aspergillus nidulans, A. fumigatus*, and *Cryptococcus gattii *[8, 13, 15, 58, 84].

*C. albicans* is the major cause of mycoses in humans, which can range from mild superficial infection of the skin to severe disseminated systemic disease. *In vitro* studies have shown that hyphae and yeast forms of *C. albicans* induce and are trapped and killed by NETs released from human neutrophils, as well as *C. neoformans* [13, 15]. Calprotectins, a calcium-binding cytoplasmic heterodimeric protein complex, were shown as the major antifungal component associated to the NETs, *in vitro* and *in vivo*. Calprotectin chelates essential metal ions such as Zn^2+^ and Mn^2+^ resulting in reduced *C. albicans *growth in subcutaneous and pulmonary infection of mice [15, 85]. Moreover, immunodepletion of calprotectin abolished the growth inhibitory activity of NETs *in vitro*, and calprotectin-deficient mice were unable to clear *C. albicans* infection [15].

The role of calprotectin against *Aspergillus* was also established in calprotectin knockout mice, which lost the ability to control the fungal infection [86]. On the contrary, an *in vitro* study using *A. fumigatus* showed that although resting conidia and germinal tube forms are able to induce NETs, the webs did not kill the fungus. The control of swollen conidia germination is achieved by phagocytosis, although NET inhibits polar growth of germ tube forms in a calprotectin-dependent manner [9].

In a human case of an invasive pulmonary aspergillosis caused by *A. nidulans *and refractory to therapy, the role of NET formation was pointed out through a successful restoration of the immune response against this fungus by gene therapy, in an 8.5-years-old boy with chronic granulomatous disease. Restoring NADPH oxidase expression after gp91^phox^ gene transfer, neutrophils defense against the conidia and hyphae forms of *A. nidulans* was reestablished in the treated patient [58]. This study unequivocally proved the role of ROS production for NET induction, at least for fungi.

Another study showed that, in a mice model of* A. fumigatus-*lung infection, NETs arise in 3-4 hours after immigrating neutrophils reach the infectious focus. Moreover, high amount of NETs against hyphae forms were observed in comparison to conidia. The presence of hydrophobin RodA, the major component of resting conidia surface, reduced NET formation. This fungal protein was identified as being an important factor to protect conidia from recognition [87], and now it seems to be an important factor to prevent triggering of NET extrusion; however, the molecular mechanism behind prevention of NET formation remains to be elucidated [88].

The encapsulated yeast *C. gatti,* is the agent of one-tenth to one-third of pulmonary cryptococcosis and meningitis worldwise. This species is distinguished from *C. neoformans* by its occurrence in trees, rather than pigeon droppings. Special diagnostic tools used to differentiate these species include the cigar-shaped yeast morphology in the host cerebrospinal fluid, agglutinating serotype, creatine assimilation, and elongated rod-shaped basidiospores [14, 84]. A study analyzing neutrophils, interaction with *C. gatti* showed an extensively NET formation *in vitro*, but without correlation with fungal killing. Moreover, comparison studies demonstrate that *C. gatti,* expressing extracellular fibrils were more resistant to neutrophil killing than capsulate mutants [14].

Whether NETs really have a role in the control of fungal infections needs to be better explored, thence it is not the only mechanism, although it means an important tool to detain infection.

## 8. NETs and Viruses

Although presently there are no data about virus capacity to directly induce NET release, the role of netosis in viral infections has been addressed for feline leukemia virus (FeLV) and influenza A virus infections [38, 63, 89].

Our group described that netosis of cat neutrophils could be modulated by the feline leukemia virus (FeLV) infection [36]. In fact, neutrophils from FeLV (−) and from asymptomatic FeLV (+) cats release NETs when stimulated with PMA or* Leishmania*. However, neutrophils from FeLV (+) symptomatic cats spontaneously release high quantities of NETs in comparison to either the neutrophils from FeLV (−) or from asymptomatic FeLV (+) cats. On the other hand, neutrophils from FeLV (+) symptomatic cats do not respond to NET-releasing stimuli. Our data suggest that netosis could be related to disease status, at least in FeLV-feline infection, and that this feature could be used as a diagnostic tool [36].

In a recent study, NET was induced in a murine model of influenza A virus (A/Puerto Rico/8/34 H1N1) pneumonitis and correlated to lesions in the alveoli and bronchioles, leading to complications of acute respiratory distress syndrome [89]. Also analyzing infection with influenza A virus (A/WSN/33/H1N1 strain) in NET-impaired mice (PAD4 knocked out) and in wild-type counterparts, NET induction was found in the bronchoalveolar lavage (BAL) of the wild-type mice, but not in the PAD4^−/−^ mice [63]. Interestingly, neutrophils obtained from lungs of wild-type mice release NET upon contact with influenza A-infected alveolar epithelial cells. However, no differences in morbidity and mortality were observed in both mice strains, ruling out the hypothesis that NET could mediate viral clearance in this model of acute infection [63].

## 9. *In Vivo* Detection of ETosis

The physiologic role of ETs can be supported by its abundance in sites of infected and noninfected models of inflammation. Accordingly, etosis has been reported in spontaneous human appendicitis [1], experimental dysentery induced by *Shigella* in rabbits [1], infections by *Streptococcus* [90], pneumonia by *Streptococcus pneumonia *[90], blood of children infected by *Plasmodium falciparum *[83], periodontitis [91], infections by *Aspergillus nidulans* [58] and by *Eimeria bovis* [16], as well as in pulmonary* Aspergillus fumigatus* infections [8], and cutaneous lesions of leishmaniasis [35]. This phenomenon was also demonstrated to be relevant in human preeclampsia [31], in small vessels vasculitis [50], in systemic lupus erythematosus [7, 50, 77], on thrombosis [92], and in psoriasis [32].

## 10. Closing Remarks

Although etosis was recently described [1], the literature concerning this new form of cell-mediated killing is growing quickly. Even though many advances have been achieved recently, the molecular mechanism underlying NET release is far from being understood. Different works have pointed out that only around 30% of the neutrophils stimulated by different stimuli dye by netosis. Many different microorganisms and molecules induce NET release, but it is unknown if netosis is triggered by common or different signaling pathways. Besides, many of the stimuli that induce NET release were formerly also described as inducers of other neutrophil functions, such as phagocytosis or chemotaxis. Thus, it is important to determine how and why some neutrophils, facing a parasite for instance, activate the netosis process, while others in the same population phagocytose the parasite. Neutrophils were short-lived cells and, presently, no markers of subpopulations are available to test if the different outcomes reflect distinct neutrophil subpopulations. Also, the maturing status of these cells could contribute for these differences, or the cytokine milieu as has been reported for polarized tumor-associated neutrophils [93].

Anyhow, netosis, or etosis in general, must be a strictly regulated process since it is a death mechanism, which released components, could participate either in antimicrobial defense and/or harm host tissues, and induce autoimmunity, where these structures are gaining increased relevance.

ETosis is proving to be a critical mechanism of host defense, offering new potential for disease control and defining new targets for intervening on infectious and also autoimmune diseases, besides grant novel tools for diagnosis [94] and/or prognosis [95].

## Supplementary Material

Video microscopy of the neutrophil-Leishmania amazonensis interaction. Human neutrophils (N) were incubated with stationary-phase (P) promastigotes (1N:5P ratio) and NETs were stained with Sytox green. The interaction was recorded during 2:55 h using an epifluorescence microscope (Zeiss AxioObserver) equipped with Colibri ilumination LED system. Frames of live Leishmania-neutrophil interaction were acquired in high resolution mode using definitive focus correction, differential interference contrast (DIC) and green fluorescent excitation filter. White arrow indicates a NET-trapped promastigote. Note that netosis visualized as a green staining is increasing over time. Bar, 5 *µ*m.

## Figures and Tables

**Table 1:** Microorganisms or molecules able to trigger extracellular traps release.

Activators	Cells	Reference
Activated endothelial cell	Neutrophil	[6]
Antiribonucleoprotein IgG	SLE Neutrophil	[7]
*Aspergillus fumigatus* (conidia or hyphae)	Neutrophil	[8, 9]
Autoantibodies (anti-LL-37/anti-HNP)	SLE Neutrophil	[10]
Calcium	HL-60 lineage, neutrophil	[11, 12]
*Candida albicans* (hyphae or yeast)	Neutrophil	[13]
*Cryptococcus gattii*	Neutrophil	[14]
*Cryptococcus neoformans*	Neutrophil	[15]
*Eimeria bovis*	Neutrophil	[16]
*Enterococcus faecalis *	Neutrophils	[17]
Equine spermatozoa	Neutrophil	[18]
*Escherichia coli*	Neutrophil, monocyte	[17, 19–21]
Glucose oxidase	Neutrophil	[22]
GM-CSF + C5a	Neutrophil	[23, 24]
GM-CSF + LPS	Neutrophil	[24]
*Haemophilus influenzae*	Neutrophil	[25, 26]
*Helicobacter pylori*	Neutrophil	[27]
Hydrogen peroxide	Neutrophil, mast cell, chicken heterophil	[22, 28, 29]
Interferon (IFN)-*α*+ C5a	Neutrophil	[23]
IFN-*γ* + C5a	Neutrophil, eosinophil	[23, 30]
IFN-*γ* + C5a, LPS or eotaxin	Eosinophil	[30]
Interleukin 5 + LPS/C5a/eotaxin	Eosinophil	[30]
Interleukin 8	Neutrophil	[1, 31]
Interleukin 23 and IL-1*β*	Mast cells	[32]
*Klebsiella pneumoniae*	Neutrophil (tissue)	[33]
*Lactococcus lactis*	Neutrophil	[34]
*Leishmania amazonensis* (promastigotes/amastigotes/lipophosphoglycan)	Neutrophil	[35]
*Leishmania amazonensis, L. donovani, L. major, L. chagasi *(promastigotes)	Neutrophil	[35–37]
Lipopolysaccharide (LPS)	Neutrophil	[1]
*Listeria monocytogenes*	Neutrophil	[20, 38]
*Mannheimia haemolytica *and leukotoxin	Neutrophil	[39]
M1 protein/M1 protein-fibrinogen complex	Neutrophil, mast cell	[40, 41]
*Mycobacterium tuberculosis, M. canettii*	Neutrophil	[42]
Nitric oxide	Neutrophil	[43, 44]
Panton-Valentine leukocidin, autolysin, and lipase	Neutrophil	[45]
Phorbol myristate acetate (PMA)	Neutrophil, mast cell, chicken heterophil	[1, 12, 28, 29]
PMA + ionomycin	Neutrophil	[17]
Platelet-activating factor	Neutrophil	[27]
Platelet TLR-4	Neutrophil	[46]
*Pseudomonas aeruginosa*	Mast cell	[28]
*Serratia marcescens*	Neutrophil	[17]
*Shigella flexneri*	Neutrophil (tissue)	[1]
*Staphylococcus aureus*	Neutrophil, mast cell	[22, 28, 45]
Statins	Neutrophil, monocytes/macrophages	[47]
*Streptococcus *(Group A–GAS)/Pilus	Neutrophil, mast cell	[34, 40, 48]
*Streptococcus dysgalactiae*	Neutrophil	[17]
*Streptococcus pneumoniae*	Neutrophil (tissue)	[49]
*Streptococcus pyogenes*	Mast cell	[28]
Syncytiotrophoblast microparticles	Neutrophil	[31]
TNF-*α*	HL-60 lineage	[11]
TNF-*α* + ANCA IgG	Neutrophil	[50]
Yeast particulate B-glucan	Neutrophil	[51]
*Yersinia enterocolitica*	Neutrophil	[52]
*δ*-Toxin from* Staphylococcus epidermidis *	Neutrophil	[53]
